# Urban and Rural Differences in Cancer Treatment Disruption Among Patients With COVID‐19: An Analysis of the US ASCO COVID‐19 in Oncology Registry

**DOI:** 10.1002/cam4.70512

**Published:** 2025-01-22

**Authors:** Yu Chen Lin, Kea Turner, Oliver T. Nguyen, Emma Hume, Marlene Camacho‐Rivera, Jessica Y. Islam

**Affiliations:** ^1^ Department of Health Outcomes and Behavior Moffitt Cancer Center Tampa Florida USA; ^2^ Department of Gastrointestinal Oncology Moffitt Cancer Center Tampa Florida USA; ^3^ Department of Community Health Sciences SUNY Downstate Health Sciences University Brooklyn New York USA; ^4^ Department of Cancer Epidemiology Moffitt Cancer Center Tampa Florida USA; ^5^ Center for Immunization and Infection Research in Cancer Moffitt Cancer Center Tampa Florida USA

**Keywords:** cancer care delivery, COVID‐19 pandemic, health disparities, health equity, rural health

## Abstract

**Introduction:**

Cancer patients in rural areas experience greater barriers to treatment access compared with patients in urban areas. There is limited research on how the COVID‐19 pandemic affected cancer treatment delivery for rural patients who were also diagnosed with COVID‐19. This study has two objectives: to assess (1) the urban–rural differences in cancer care and (2) the predictors of cancer treatment delay or discontinuation (TDD) among patients diagnosed with COVID‐19.

**Methods:**

We used data from the American Society of Clinical Oncology Survey on COVID‐19 in Oncology Registry (March 2020–September 2022), which included cancer patients with test‐confirmed SARS‐CoV‐2 infection (*N* = 3797). Data included patient sociodemographic characteristics, COVID‐19 diagnosis information, cancer clinical characteristics, and changes to cancer treatment. Cancer TDD was defined as any scheduled treatment by more than 2 weeks. Rurality was examined through both patient residence and oncology practice. We computed adjusted prevalence ratios (aPRs) using multivariable Poisson regressions to assess predictors of cancer TDD in urban and rural areas.

**Results:**

During the study period, 44.1% of patients with COVID‐19 experienced either cancer treatment delay or discontinuation and 5.7% experienced cancer treatment discontinuation. Controlling for other factors, receiving care in a rural oncology practice was associated with cancer TDD (aPR: 1.25, 95% CI: 1.01–1.55). Differences in cancer TDD were not found across rurality of patient residence. Among rural patients (*N* = 582), Hispanic/Latinx cancer patients had greater prevalence of cancer TDD (aPR: 1.55, 95% CI: 1.04–2.33) compared with non‐Hispanic White cancer patients.

**Conclusion:**

Our findings can be used to inform programs and policies to minimize the impact of future public health emergencies on cancer care delivery in rural areas. Additional research is needed to explore potential differences in cancer care delivery across urban and rural oncology practices and patients.

## 1 Introduction

Rural cancer patients in the United States experience lower quality of care and worse patient outcomes compared with urban cancer patients. Individuals in rural areas have higher cancer mortality rates, particularly for cancers that are preventable and treatable [1, 2, 3]. Cancer patients in rural areas are more likely to experience delays in cancer diagnosis and cancer treatment, miss appointments, and lack access to genomic testing [4, 5, 6, 7, 8, 9]. There are several factors that may contribute to the urban–rural disparities in cancer treatment. At the patient level, individuals in rural areas are more likely to have limited economic resources, lack health insurance, and travel a greater distance to receive care [10, 11]. At the practice level, rural practices are more likely to be smaller and have fewer specialized providers compared with urban practices [12, 13, 14].

The COVID‐19 pandemic further exacerbated these disparities in urban–rural cancer care. Rural areas experienced barriers to navigating the COVID‐19 pandemic, including lower COVID‐19 vaccination rates, fewer healthcare resources (e.g., intensive care unit [ICU] beds, healthcare workers), and a greater proportion of residents at risk for adverse COVID‐19 outcomes (e.g., older adults) compared with urban areas [15, 16, 17, 18]. The unique cancer care delivery challenges experienced by rural areas may explain disruptions to care, such as greater delays in cancer screenings [19]. System and policy changes during the pandemic, such as the reallocation of healthcare resources toward combatting the pandemic and the cancelation of elective procedures, further contributed to the burdens of rural hospitals [20]. The COVID‐19 pandemic also posed a challenge to cancer patients due to the unique interplay between the two conditions, in which related comorbidities and disruptions to timely care can result in adverse outcomes in both conditions [21, 22]. It is important to examine how the pandemic has affected urban and rural cancer care delivery differently.

Previous studies have examined outcomes in cancer patients with concurrent COVID‐19 infection. However, these studies are often limited in sample size, focused on COVID‐19 outcomes rather than cancer‐related outcomes, or were not focused on assessing urban–rural disparities [23, 24, 25, 26, 27, 28]. Other studies that have examined cancer care delivery were not focused on assessing urban–rural disparities [23, 26, 27, 28]. To address this gap, this study has two objectives: to assess (1) the urban–rural differences in cancer care and (2) the predictors of cancer treatment delay or discontinuation (TDD) among patients diagnosed with COVID‐19.

## 2 Methods

### 2.1 Data Collection and Study Population

The American Society of Clinical Oncology (ASCO) Survey on COVID‐19 in Oncology Registry (ASCO Registry) was designed to collect information about the impact of the COVID‐19 pandemic on patient outcomes and cancer care delivery [29]. The cohort study followed individuals diagnosed with COVID‐19 undergoing active cancer treatment who were receiving care in community and academic oncology practices in the United States. Data for the current study were abstracted from March 2020 to September 2022. Data were collected from 69 oncology practices including nonacademic hospitals or health systems (54%), academic institutions (23%), and physician‐owned or independent clinics (23%). Most (90%) oncology practices were located in urban areas, while 10% were located in rural areas. Oncology practices were located in four census regions: Northeast (17%), Midwest (32%), South (36%), and West (15%). The ASCO Registry was approved by the Western Institutional Review Board.

Patients at participating oncology practices were eligible to be included in the ASCO Registry if they met the following criteria: (1) received test‐confirmed COVID‐19 diagnosis and (2) is concurrently undergoing cancer treatment, including: (a) patient recently diagnosed with cancer and is undergoing cancer staging and/or initial cancer treatment; (b) patient with clinically evident cancer; (c) patient within 1 year of having active cancer, including cancer‐free patients undergoing any form of adjuvant therapy (e.g., hormone treatments) after a surgical procedure; or (d) patient with clinically evident cancer who is receiving supportive care only. For the present analysis, we excluded individuals aged ≤ 18 years and those who died within 30 days of their COVID‐19 diagnosis. A total of 3797 patients met the study criteria. Data were extracted from patients' electronic health records and entered into REDCap, a secure web‐based platform, managed by ASCO/CancerLinQ [30, 31]. Oncology practices that participated in the registry after it had begun could enter data retrospectively for eligible patients.

### 2.2 Measures

#### 2.2.1 Cancer Treatment Delay or Discontinuation

Cancer TDD was the main outcome of interest, determined by the question: “Which of the following describes how the patient's treatment plan was modified at or immediately after COVID‐19 diagnosis?” [32] Response options included: “patient received on schedule or within 14 days”; “patient receipt of therapy or surgery was delayed at least 14 days from initial treatment date”; or “patient receipt of therapy or surgery was discontinued or canceled with no plans of restart.” [32] Consistent with previous studies examining cancer patients with concurrent COVID‐19 diagnosis [26, 27, 28], we defined patients with TDD as those who had cancer treatments delayed by 14 days or more from the initially scheduled treatment date. Types of cancer treatments included surgery, radiation therapy, drug‐based therapies, and transplant or cellular therapy. Patients were included if they had a scheduled cancer treatment within 0–6 weeks of their COVID‐19 diagnosis. For patients undergoing many cancer treatments, changes to the planned treatment were provided for each treatment type. Clinics also reported the reason for TDD by treatment type.

#### 2.2.2 Sociodemographic Factors and Health History

The main exposure of interest was rural status based on the patient's residence zip code, assigned using the 2010 US Department of Agriculture's (USDA) Rural–Urban Commuting Area (RUCA) codes [33]. RUCA codes at the census tract level were mapped to zip codes and considered population density, urbanization, and commuting patterns in determining the rurality of the area [33]. Urban patients included those living in metropolitan areas (RUCA codes 1–3); rural patients included those living in nonmetropolitan areas (RUCA codes 4–10). Race and ethnicity were recorded using the following categories: non‐Hispanic White (NH‐White), NH‐Black, NH‐Asian, NH‐American Indian or Alaska Native (NH‐AI/AN), and Hispanic/Latinx. Additional patient demographic characteristics included age group at COVID‐19 diagnosis, sex at birth, and census region of patient residence. Health history information included tobacco use, body mass index (BMI), and number of comorbidities. Several area‐level social determinants of health measures were computed by matching the patient's residence zip code to the Agency of Healthcare Quality's Social Determinants of Health Database, specifically zip code‐level data derived from the Census Bureau's American Community Survey 2014–2018 5‐year estimates, and were provided by ASCO to the research team in quartiles to prevent the deidentification of patient data. These area‐level measures included median household income, percentage of population with only a high school degree, and percentage of population under age 65 without health insurance.

#### 2.2.3 Cancer and COVID‐19 Information

The ASCO Registry included various patient‐level cancer and COVID‐19 information. Similar to the rural status for patient residence, rural status of the oncology practice was classified based on the practice's zip code according to the USDA RUCA codes described previously. Cancer information included: cancer type (based on the International Statistical Classification of Diseases and Related Health Problems, Tenth Revision [ICD‐10] diagnosis codes), extent of cancer, cancer status, and type of treatment scheduled when diagnosed with COVID‐19. ECOG performance status, a measure of the extent to which the disease affects a patient's activities of daily living, was also captured. COVID‐19 information included whether the patient developed any COVID‐related complications and COVID‐19 severity (measured by whether the patient required mechanical ventilation or nonmechanically ventilated hospitalization and ICU admission). We excluded cancer patients who died from COVID‐19 within 30 days of being diagnosed.

#### 2.2.4 Time

COVID‐19 case surges that may be associated with cancer TDD were accounted for using a time variable of when the patient received their COVID‐19 diagnosis, categorized into five time periods that corresponded to periods of low or high COVID‐19 cases and the various dominant COVID‐19 strains: first wave (March–June 2020), second wave (July–November 2020), third wave (December 2020–March 2021), fourth wave (April 2021–February 2022), and fifth wave (March–September 2022) [34, 35]. These time periods may also be associated with differential rates of COVID‐19 vaccination and treatment [35].

### 2.3 Statistical Analyses

Among patients with scheduled cancer treatment within 0–6 weeks at the time of their COVID‐19 diagnosis who met our study criteria (*N* = 3797), we summarized patient characteristics and compared the proportion of each characteristic across the rural status of patient residence using Pearson chi‐squared tests. To evaluate the predictors of patients experiencing TDDs among urban and rural patients, we computed adjusted prevalence ratios (aPRs) and 95% confidence intervals (95% CIs) using multivariable Poisson regressions with a log link function [36, 37, 38], adjusting for sociodemographic characteristics, health history, area‐level characteristics, and cancer and COVID‐19 information described previously. Standard errors were robust and clustered at the oncology practice level to account for the nonindependence of patients within clusters. A complete case approach was used to account for missing data (approximately 9.7% of the sample) in the multivariable regressions. Finally, we summarized the primary reason for cancer TDD by treatment type and compared the proportions between the rural status of patient residence using Fisher's exact test due to small, expected frequencies.

A two‐sided *p* < 0.05 was deemed to be statistically significant. All analyses were performed in Stata SE v.17 (StataCorp, College Station, TX, USA). This study was reviewed by the Moffitt Cancer Center Institutional Review Board of Record, Advarra, and deemed exempt from obtaining informed consent. Reporting of this study complied with the Strengthening the Reporting of Observational Studies in Epidemiology (STROBE) statement [39].

## 3 Results

### 3.1 Sample Characteristics

Overall, the sample included 3797 individuals with scheduled cancer treatment within 0–6 weeks of their COVID‐19 diagnosis (Table 1). Most individuals (84.6%) resided in urban rather than rural areas (15.3%); one individual had missing information on their rural status. The age distribution of the sample included 18–34 years (3.7%), 35–49 years (14.0%), 50–64 years (35.8%), and 65 years or older (46.5%). Most participants identified as female (60.0%) and the race/ethnicity of the sample included 68.1% NH‐White, 12.6% NH‐Black, 11.6% Hispanic/Latinx, 4.2% NH‐Asian, and 3.5% NH‐AI/AN. Most individuals had never smoked (50.3%). Patients' weight included underweight (2.3%), healthy weight (25.7%), overweight (31.9%), or obese (40.2%). About half of patients had 1–2 comorbidities (48.3%); fewer patients had 3–4 comorbidities (10.2%), 5 or more comorbidities (3.5%), or no comorbidities (38.0%). Quartiles of area‐level characteristics, including median household income, percentage of population with only a high school degree, and percentage of population under age 65 without health insurance, were also described in Table 1.

**TABLE 1:** Characteristics of cancer patients with scheduled cancer treatment diagnosed with COVID‐19, stratified by rural status of patient residence.

Characteristics	Frequency (%)			*p* ^a^
	Overall (*N* = 3797)	Urban (*N* = 3214)	Rural (*N* = 582)	
Sociodemographic				
Age group				
18–34	139 (3.7)	120 (3.7)	19 (3.3)	0.053
35–49	533 (14.0)	467 (14.5)	66 (11.3)	
50–64	1359 (35.8)	1160 (36.1)	199 (34.2)	
≥ 65	1765 (46.5)	1466 (45.6)	298 (51.2)	
Race/ethnicity				
White, non‐Hispanic	2584 (68.1)	2105 (65.5)	478 (82.1)	< 0.001
Black, non‐Hispanic	477 (12.6)	438 (13.6)	39 (6.7)	
Hispanic/Latinx	441 (11.6)	405 (12.6)	36 (6.2)	
Asian, non‐Hispanic	160 (4.2)	144 (4.5)	16 (2.7)	
American Indian/Alaskan Native, non‐Hispanic	134 (3.5)	121 (3.8)	13 (2.2)	
Sex				
Male	1518 (40.0)	1286 (40.0)	232 (39.9)	0.946
Female	2279 (60.0)	1928 (60.0)	350 (60.1)	
Census region				
Northeast	488 (12.9)	481 (15.0)	7 (1.2)	< 0.001
Midwest	1214 (32.0)	954 (29.7)	260 (44.7)	
South	1779 (46.9)	1513 (47.1)	266 (45.7)	
West	315 (8.3)	266 (8.3)	49 (8.4)	
Health history				
Tobacco use				
Current smoker	352 (9.3)	299 (9.3)	53 (9.1)	0.358
Former smoker	1428 (37.6)	1191 (37.1)	236 (40.6)	
Never smoked	1908 (50.3)	1628 (50.7)	280 (48.1)	
Unknown	109 (2.9)	96 (3.0)	13 (2.2)	
Body mass index				
Underweight	84 (2.3)	71 (2.3)	13 (2.3)	0.886
Healthy weight	952 (25.7)	803 (25.6)	148 (25.9)	
Overweight	1181 (31.9)	992 (31.6)	189 (33.0)	
Obesity	1491 (40.2)	1269 (40.5)	222 (38.8)	
Number of comorbidities^b^				
None	1442 (38.0)	1240 (38.6)	202 (34.7)	0.176
1–2 comorbidities	1833 (48.3)	1537 (47.8)	295 (50.7)	
3–4 comorbidities	388 (10.2)	320 (10.0)	68 (11.7)	
5+ comorbidities	134 (3.5)	117 (3.6)	17 (2.9)	
Area‐level characteristics (of patient's residence zip code)^c^				
Median household income				
<$43,125	630 (17.9)	458 (15.0)	172 (37.9)	< 0.001
$43,125–$54,047	836 (23.8)	651 (21.3)	185 (40.7)	
$54,048–$68,446	931 (26.5)	846 (27.6)	85 (18.7)	
≥ $68,446.50	1118 (31.8)	1106 (36.1)	12 (2.6)	
Percentage of population with only a high school degree				
< 25.5%	1182 (33.6)	1155 (37.7)	27 (5.9)	< 0.001
25.5%–33.7%	1248 (35.5)	1078 (35.2)	170 (37.2)	
33.8%–41.2%	859 (24.4)	693 (22.6)	166 (36.3)	
≥ 42.2%	229 (6.5)	135 (4.4)	94 (20.6)	
Percentage of population under age 65 without health insurance				
< 4.8%	488 (13.9)	461 (15.1)	27 (5.9)	< 0.001
4.8%–8.8%	1063 (30.2)	970 (31.7)	93 (20.4)	
8.9%–14.7%	1211 (34.4)	990 (32.3)	221 (48.4)	
≥ 14.8%	756 (21.5)	640 (20.9)	116 (25.4)	
Cancer information				
Patient experienced treatment delay				
No	2121 (55.9)	1765 (54.9)	355 (61.0)	0.007
Yes	1676 (44.1)	1449 (45.1)	227 (39.0)	
Patient experienced treatment discontinuation				
No	3597 (94.7)	3034 (94.4)	562 (96.6)	0.032
Yes	200 (5.3)	180 (5.6)	20 (3.4)	
Rural status of oncology practice				
Urban	3579 (94.3)	3178 (98.9)	400 (68.7)	NC^d^
Rural	218 (5.7)	36 (1.1)	182 (31.3)	
Cancer treatment type received or scheduled to receive (multiple may apply)				
Surgery	215 (5.7)	186 (5.8)	29 (5.0)	0.440
Radiation therapy	360 (9.5)	300 (9.3)	60 (10.3)	0.460
Drug‐based therapy	3440 (90.6)	2904 (90.4)	535 (91.9)	0.233
Transplant or cellular therapy	30 (0.8)	23 (0.7)	7 (1.2)	0.222
Cancer type^e^				
Breast	1078 (28.4)	921 (28.7)	157 (27.0)	0.588
Hematologic/blood	883 (23.3)	746 (23.2)	136 (23.4)	
Digestive/gastrointestinal	505 (13.3)	419 (13.0)	86 (14.8)	
Lung	374 (9.9)	309 (9.6)	65 (11.2)	
Genitourinary	332 (8.7)	287 (8.9)	45 (7.7)	
Gynecological	169 (4.5)	140 (4.4)	29 (5.0)	
Other	456 (12.0)	392 (12.2)	64 (11.0)	
Extent of cancer				
Local	809 (21.3)	689 (21.4)	120 (20.6)	0.885
Regional	406 (10.7)	345 (10.7)	61 (10.5)	
Metastatic	1394 (36.7)	1169 (36.4)	225 (38.7)	
Cancer‐free but receiving adjuvant therapy	222 (5.8)	190 (5.9)	32 (5.5)	
Unknown	966 (25.4)	821 (25.5)	144 (24.7)	
Cancer status				
Progressing	480 (12.6)	395 (12.3)	85 (14.6)	0.387
Stable	1213 (31.9)	1024 (31.9)	189 (32.5)	
Responding to treatment	267 (7.0)	226 (7.0)	41 (7.0)	
Unknown	1837 (48.4)	1569 (48.8)	267 (45.9)	
ECOG performance status				
0	1276 (33.6)	1113 (34.6)	163 (28.0)	< 0.001
1	1221 (32.2)	1013 (31.5)	208 (35.7)	
2	381 (10.0)	326 (10.1)	55 (9.5)	
3+	134 (3.5)	125 (3.9)	9 (1.5)	
Unknown	785 (20.7)	637 (19.8)	147 (25.3)	
Cancer diagnosis year				
2010 or earlier	201 (5.3)	173 (5.4)	28 (4.8)	0.795
2011–2019	1735 (45.7)	1463 (45.5)	271 (46.6)	
2020–2022	1859 (49.0)	1577 (49.1)	282 (48.5)	
COVID‐19 information				
Patient developed any COVID‐19 complications^f^				
No	3201 (84.3)	2697 (83.9)	503 (86.4)	0.125
Yes	596 (15.7)	517 (16.1)	79 (13.6)	
COVID‐19 severity				
Uncomplicated	2769 (72.9)	2328 (72.4)	440 (75.6)	0.214
Hospitalized, nonmechanically ventilated	892 (23.5)	766 (23.8)	126 (21.6)	
ICU admission, nonmechanically ventilated	90 (2.4)	77 (2.4)	13 (2.2)	
Mechanically ventilated	46 (1.2)	43 (1.3)	3 (0.5)	
COVID‐19 case surge waves				
First wave (March–June 2020)	155 (4.1)	147 (4.6)	8 (1.4)	< 0.001
Second wave (July–November 2020)	749 (19.7)	629 (19.6)	120 (20.6)	
Third wave (December 2020–March 2021)	983 (25.9)	830 (25.8)	152 (26.1)	
Fourth wave (April 2021–February 2022)	1316 (34.7)	1081 (33.6)	235 (40.4)	
Fifth wave (March–September 2022)	594 (15.6)	527 (16.4)	67 (11.5)	

^a^ *p*‐values were computed using Pearson's chi‐squared test to compare the proportions of each characteristic across the rural status of patient residence.

^b^ Potential comorbidities included alcoholism, chronic supplemental oxygen needed, cirrhosis, congestive heart failure, coronary artery disease, dementia, diabetes, hepatitis, history of solid organ transplant, HIV/AIDS, hypertension, immunosuppressed due to noncancer‐related treatment (defined as outpatient use of systemic corticosteroids [≥ 10 mg/day prednisone], use of chemotherapy, or use of immunosuppressive agents for solid organ transplant or for an autoimmune disease), inflammatory bowel disease, pulmonary disease, renal condition, and systemic autoimmune disease.

^c^ Area‐level characteristics were computed by matching the patient's residence zip code to the 2018 American Community Survey data and were provided by ASCO to the research team in the quartiles shown to prevent the deidentification of patient data.

^d^ Could not be computed.

^e^ Hematologic/blood cancers included leukemia, non‐Hodgkin lymphoma, other lymphomas, and multiple myeloma. Digestive/gastrointestinal cancers included colon, rectal, pancreatic, stomach, small intestine, gallbladder, and liver cancers. Gynecological cancers included endometrial, cervical, and ovarian cancers. Genitourinary cancers included bladder, kidney, and prostate cancers. Other cancers included unspecified cancer types and cancer types with < 4% prevalence in the analytical dataset, such as head and neck, skin, neurological, and musculoskeletal cancers.

^f^ COVID‐19 complications included bleeding, disseminated intravascular coagulation, sepsis, acute respiratory distress syndrome, pneumonitis, pulmonary embolism, respiratory failure, cardiac arrhythmia, cerebrovascular accident, congestive heart failure, deep venous thrombosis, myocardial infarction, acute hepatic injury, bowel perforation, peritonitis, acute renal failure, encephalopathy, and seizures.

More than two‐fifths of individuals (44.1%) experienced cancer TDD, with 5.3% of individuals experiencing treatment discontinuation (Table 1). In the unadjusted comparison, urban patients had a higher prevalence of experiencing TDD compared to rural patients (45.1% vs. 39.0%; *p* = 0.007) and had a higher prevalence of experiencing treatment discontinuation (5.6% vs. 3.4%; *p* = 0.032). Most individuals received cancer care in an urban practice (94.3%) rather than a rural practice (5.7%). Patients were scheduled to receive surgery (5.7%), radiation therapy (9.5%), drug‐based therapy (90.6%), or transplant/cellular therapy (0.8%). About one‐quarter of patients were diagnosed with breast cancer (28.4%) or hematologic/blood cancers (23.3%), and more than one‐third of patients (36.7%) had metastatic cancer. Most individuals (72.9%) experienced COVID‐19 that did not require hospitalization or medical interventions, and 84.3% of individuals did not develop any COVID‐19 complications (e.g., bleeding, sepsis, respiratory failure). Less than a quarter of patients (23.5%) experienced hospitalizations. Among hospitalized patients, 2.4% experienced an ICU admission that did not require mechanical ventilation, while 1.2% experienced an ICU admission that required mechanical ventilation.

### 3.2 Predictors of Cancer Treatment Delay or Discontinuation

After adjusting for other factors shown in Table 1, patient residence was no longer associated with prevalence of cancer TDD (aPR: 0.88, 95% CI: 0.71–1.09; *p* = 0.245) (Table 2; not all variables were shown for brevity). However, receiving care in a rural oncology practice was associated with greater prevalence of patients experiencing TDD (aPR: 1.25, 95% CI: 1.01–1.55; *p* = 0.037), particularly for patients residing in urban areas who sought care at a rural practice (aPR: 1.41, 95% CI: 1.11–1.80; *p* = 0.005). Overall, NH‐Black patients (aPR: 1.17, 95% CI: 1.04–1.31; *p* = 0.009) and Hispanic/Latinx patients (aPR: 1.14, 95% CI: 1.03–1.27; *p* = 0.014) had higher prevalence of TDD compared to NH‐White patients. Rural patients with an ECOG performance status of 3 or greater (i.e., individuals capable of limited self‐care or were completely disabled) had higher prevalence of TDD (aPR: 1.86, 95% CI: 1.11–3.11; *p* = 0.018). Other factors associated with significantly higher overall prevalence of TDD included older age (50 years or older); receiving surgery, radiation therapy, or transplant/cellular therapy compared to not receiving that type of treatment; having digestive/gastrointestinal cancers; having regional or metastatic cancers or being cancer‐free but receiving adjuvant therapy; and having a recent cancer diagnosis. Supporting Information—Table S1 shows the same model presented in Table 2 with the full regression results of all included covariates.

**TABLE 2:** Predictors of cancer treatment delay or discontinuation among urban and rural cancer patients with COVID‐19.

Variables	Outcome: cancer treatment delay or discontinuation		
	aPR (95% CI)^a^		
	Overall	Urban	Rural
Sociodemographic			
Rural status of patient residence			
Urban	Ref.	—	—
Rural	0.88 (0.71–1.09)	—	—
Age group			
18–34	Ref.	Ref.	Ref.
35–49	1.13 (0.95–1.35)	1.20 (0.99–1.45)	0.71 (0.40–1.24)
50–64	1.27* (1.06–1.53)	1.31** (1.09–1.57)	1.00 (0.60–1.66)
≥ 65	1.21* (1.00–1.46)	1.21* (1.01–1.46)	1.32 (0.76–2.29)
Race/ethnicity			
White, non‐Hispanic	Ref.	Ref.	Ref.
Black, non‐Hispanic	1.17** (1.04–1.31)	1.14* (1.02–1.27)	1.41 (0.94–2.11)
Hispanic/Latinx	1.14* (1.03–1.27)	1.12* (1.02–1.23)	1.55* (1.04–2.33)
Asian, non‐Hispanic	0.84* (0.73–0.97)	0.88 (0.74–1.04)	0.34* (0.12–0.95)
American Indian/Alaskan Native, non‐Hispanic	0.89 (0.67–1.18)	0.84 (0.64–1.11)	1.31 (0.63–2.71)
Sex			
Male	Ref.	Ref.	Ref.
Female	1.03 (0.96–1.11)	1.03 (0.95–1.11)	1.00 (0.78–1.28)
Census region			
Northeast	Ref.	Ref.	Ref.
Midwest	0.91 (0.67–1.24)	0.92 (0.67–1.25)	0.79 (0.35–1.76)
South	1.08 (0.80–1.46)	1.07 (0.79–1.46)	1.08 (0.53–2.21)
West	1.06 (0.69–1.62)	1.14 (0.74–1.74)	0.43 (0.15–1.22)
Cancer information			
Rural status of oncology practice			
Urban	Ref.	Ref.	Ref.
Rural	1.25* (1.01–1.55)	1.41** (1.11–1.80)	0.99 (0.71–1.38)
Cancer treatment type received or scheduled to receive (Ref. = did not receive or was not scheduled to receive that treatment type)			
Surgery	1.77*** (1.49–2.09)	1.71*** (1.41–2.07)	3.03*** (1.60–5.71)
Radiation therapy	1.13* (1.02–1.25)	1.07 (0.97–1.19)	2.02*** (1.39–2.93)
Drug‐based therapy	1.09 (0.92–1.30)	1.12 (0.95–1.33)	1.10 (0.70–1.71)
Transplant or cellular therapy	1.72*** (1.37–2.16)	1.50** (1.14–1.97)	3.41*** (2.18–5.34)
Cancer type			
Breast	Ref.	Ref.	Ref.
Hematologic/blood	1.34 (0.98–1.83)	1.27 (0.92–1.76)	2.35** (1.27–4.33)
Digestive/gastrointestinal	1.26*** (1.12–1.42)	1.24*** (1.10–1.40)	1.47* (1.08–2.01)
Lung	1.07 (0.95–1.21)	1.08 (0.93–1.25)	0.94 (0.50–1.75)
Genitourinary	0.93 (0.81–1.07)	0.93 (0.80–1.09)	0.91 (0.49–1.72)
Gynecological	1.14 (0.95–1.36)	1.14 (0.96–1.35)	1.19 (0.65–2.17)
Other	0.93 (0.78–1.10)	0.97 (0.82–1.15)	0.51* (0.30–0.88)
Extent of cancer			
Local	Ref.	Ref.	Ref.
Regional	1.25*** (1.12–1.39)	1.20** (1.07–1.35)	1.59* (1.07–2.35)
Metastatic	1.30*** (1.13–1.49)	1.27** (1.09–1.48)	1.44* (1.01–2.04)
Cancer‐free but receiving adjuvant therapy	0.63** (0.46–0.87)	0.63** (0.45–0.87)	0.60 (0.23–1.56)
Unknown	0.92 (0.66–1.28)	0.93 (0.66–1.31)	0.73 (0.41–1.28)
Cancer status			
Progressing	Ref.	Ref.	Ref.
Stable	0.98 (0.88–1.09)	0.97 (0.86–1.10)	0.88 (0.65–1.20)
Responding to treatment	0.92 (0.77–1.11)	0.93 (0.75–1.15)	0.73 (0.42–1.26)
Unknown	1.01 (0.82–1.25)	1.05 (0.85–1.29)	0.64 (0.40–1.04)
ECOG performance status			
0	Ref.	Ref.	Ref.
1	1.07 (0.97–1.19)	1.06 (0.95–1.19)	1.07 (0.80–1.42)
2	1.02 (0.90–1.16)	1.00 (0.87–1.14)	1.00 (0.71–1.41)
3+	1.19* (1.01–1.41)	1.16 (0.99–1.37)	1.86* (1.11–3.11)
Unknown	1.05 (0.91–1.20)	1.05 (0.90–1.23)	0.83 (0.61–1.13)
Cancer diagnosis year			
2010 or earlier	Ref.	Ref.	Ref.
2011–2019	1.07 (0.90–1.28)	1.09 (0.91–1.31)	0.80 (0.51–1.26)
2020–2022	1.25** (1.07–1.46)	1.29** (1.10–1.52)	0.87 (0.55–1.37)
*N*	3428	2982	446

^a^ Adjusted prevalence ratios and 95% confidence intervals were computed using a multivariable Poisson regression with a log link function, adjusting for sociodemographic characteristics (rural status of patient residence, age group, race/ethnicity, sex, census region), health history (tobacco use, BMI, number of comorbidities), area‐level characteristics (median household income, population with only a high school degree, population under age 65 without health insurance), cancer information (rural status of oncology practice, treatment type, cancer type, extent of cancer, cancer status, ECOG performance status, diagnosis year), and COVID‐19 information (complications, severity, case surge wave time period). Standard errors were robust and clustered by the oncology practice. Not all variables were shown for brevity.

* *p* < 0.05.

** *p* < 0.01.

*** *p* < 0.001.

Overall, patients' COVID‐19 disease was the predominant reason for TDD for surgery (93.9%), radiation therapy (85.8%), drug‐based therapy (84.5%), and transplant/cellular therapy (82.6%) (results not shown in tables). Reasons for TDD were similar across urban and rural areas for radiation therapy, drug‐based therapy, and transplant/cellular therapy. Urban patients were more likely to experience surgery TDD due to the patients' COVID‐19 disease or other/unknown reasons compared with rural patients, who were more likely to experience surgery TDD due to the patient's progressive or recurrent disease (*p* = 0.029).

## 4 Discussion

The aim of this study was to assess the urban–rural differences in cancer care and the predictors of cancer TDD among patients diagnosed with COVID‐19. To our knowledge, this is the first large‐scale, multicenter study examining urban and rural disparities in cancer TDD among patients diagnosed with COVID‐19. We found that patients receiving care in rural oncology practices had higher prevalence of cancer TDD compared with patients receiving care in urban oncology practices. Consistent with prior research, our findings suggest that the COVID‐19 pandemic may have affected rural and urban practices differently. Prepandemic, rural hospitals faced significant financial problems and high risk of closure due to decreasing rural populations [20]. During the pandemic, system and policy changes, such as reallocation of healthcare resources to combatting the pandemic and the cancelation of elective procedures, further exacerbated the pre‐existing challenges faced by rural hospitals [20]. Rural practices were also less equipped to manage the surge of patients while maintaining timely cancer care due to difficulty with obtaining personal protective equipment, staffing shortages, and reduced delivery of cancer screening services [20, 40, 41].

Similarities in cancer treatment disparities and the risk of COVID‐19 (e.g., socioeconomic status, health comorbidities, and access to health care) have been described elsewhere [42]. Our findings can be used to inform programs and policies to minimize the impact of future public health emergencies on cancer care delivery in rural areas. These policies can include structural changes (e.g., efforts to eliminate discrimination and stigmatization against historically disenfranchised communities) and system changes (e.g., expansion of cancer screening and patient navigation programs) to ensure equity in cancer care [43]. In addition, community outreach and engagement can also help communities prepare for and appropriately respond to public health emergencies [44].

We did not find differences in cancer TDD based on the rural status of patient's residence, though we did find that rural patients with high ECOG performance status had higher prevalence of TDD, which is consistent with rural patients having to travel farther distances to receive care [11]. Prior research has noted that rural cancer patients were less likely to access telemedicine compared with urban cancer patients during the pandemic [45]. One study also showed that rural breast cancer patients experienced longer treatment initiation delays compared with urban patients during the pandemic [46]. Additional research is needed to understand how the pandemic affected cancer care delivery for rural residents and to reconcile the differences in findings for rural residents and rural oncology practices.

Overall, and within rural areas, Hispanic/Latinx cancer patients diagnosed with COVID‐19 had higher prevalence of cancer TDD compared with NH‐White patients. This finding is consistent with prior research demonstrating that Hispanic/Latinx cancer patients were more likely to experience treatment delays during the pandemic [26, 27, 47]. Prior studies have shown that Hispanic/Latinx cancer patients experience disparities in cancer care, including access to screening, time to follow‐up after positive screening results, time‐to‐treatment initiation, and stage of diagnosis [48, 49, 50]. Multilevel factors contribute to the ethnic disparities in cancer care delivery, such as social determinants of health, lack of culturally relevant cancer control interventions, and lack of linguistically competent care [51, 52, 53, 54, 55]. While our study accounted for some factors that may explain ethnic disparities such as area‐level social determinants of health, other measures such as language barriers were not available. In‐depth qualitative explorations of barriers and facilitators to cancer care access during the pandemic among Hispanic/Latinx cancer patients should be prioritized for future work.

Cancer patients diagnosed with COVID‐19 is an important patient population to study due to the interplay of comorbidities and the disruptions in care that could lead to adverse outcomes in both conditions [20, 21], including higher risk of hospitalization, ICU admission, and death [56, 57, 58]. Our findings showed that the overwhelming reason for cancer TDD across all treatment types was the patient's COVID‐19 disease. The downstream consequences of the impact of the pandemic on interruptions to cancer care should be monitored in future work as they may lead to disparate cancer deaths and adverse outcomes [59].

### 4.1 Limitations

This study should be considered in light of the following limitations. First, hospitals during the COVID‐19 pandemic recommended an isolation policy for patients diagnosed with COVID‐19, which may overlap with the delay in scheduled cancer treatment. However, our study included patients with treatments scheduled within 0–6 weeks of the patient's COVID‐19 diagnosis. Future studies with access to more detailed data on cancer TDD can tease apart the impact of the isolation period. Second, participation in the ASCO Registry was voluntary, and findings from this study on patients and oncology practices in the ASCO Registry may not be generalizable to the overall population. Third, the ASCO Registry did not include many practice‐level characteristics that may be important in explaining cancer TDD between urban and rural practices. Fourth, while our study included area‐level measures of socioeconomic status, the ASCO Registry had limited individual‐level measures of socioeconomic status, such as household income and health insurance access, that may be important to consider in the future when exploring differences in cancer TDD across urban and rural areas. Nonetheless, this is the first study we are aware of that assessed cancer TDD across urban and rural residents and oncology practices among patients with COVID‐19.

## 5 Conclusion

Our study found that patients receiving care in a rural oncology practice had higher prevalence of cancer TDD compared with patients receiving care in an urban oncology practice. Differences in cancer TDD were not found across patients residing in urban and rural areas. Rural Hispanic/Latinx cancer patients were more likely to experience cancer TDD. To contextualize these findings, future qualitative studies are needed to explore potential differences in cancer care during the COVID‐19 pandemic across rural and urban practices and among Hispanic/Latinx cancer patients.

## Author Contributions

**Yu Chen Lin:** data curation (supporting), formal analysis (supporting), writing – original draft (equal). **Kea Turner:** conceptualization (supporting), writing – original draft (equal). **Oliver T. Nguyen:** writing – review and editing (equal). **Emma Hume:** writing – review and editing (equal). **Marlene Camacho‐Rivera:** writing – review and editing (equal). **Jessica Y. Islam:** conceptualization (lead), data curation (lead), formal analysis (lead), funding acquisition (lead), investigation (lead), methodology (lead), supervision (lead), writing – original draft (equal).

## Ethics Statement

This study was reviewed by the Moffitt Cancer Center Institutional Review Board of Record, Advarra, and deemed exempt from obtaining informed consent. The ASCO Registry was approved by the Western Institutional Review Board.

## Conflicts of Interest

JYI received consulting fees from Flatiron Health Inc. All other authors declare no conflicts of interest.

## Supporting information


Data S1:

## Data Availability

Data from the ASCO Survey on COVID‐19 in Oncology Registry can be requested directly from ASCO: https://society.asco.org/covid‐resources/asco‐registry.
