# Supplementary material for: Urban and Rural Differences in Cancer Treatment Disruption Among Patients With COVID‐19: An Analysis of the US ASCO COVID‐19 in Oncology Registry
Source: Cancer Med. 2025 Jan 22;14(2):e70512. doi: 10.1002/cam4.70512 (PMC11754248; doi:10.1002/cam4.70512)
Supplement: Supplementary file 1 — Data S1: [file CAM4-14-e70512-s001.docx]

| **Table S1. Predictors of cancer treatment delay or discontinuation among urban and rural cancer patients with COVID-19, full.** | | | |
| --- | --- | --- | --- |
|  | **Outcome: Cancer treatment delay or discontinuation** | | |
|  | **aPR (95% CI)^a^** | | |
| **Variables** | **Overall** | **Urban** | **Rural** |
| **Sociodemographic** |  |  |  |
| Rural status of patient residence |  |  |  |
| Urban | Ref. | -- | -- |
| Rural | 0.88 (0.71-1.09) | -- | -- |
| Age group |  |  |  |
| 18-34 | Ref. | Ref. | Ref. |
| 35-49 | 1.13 (0.95-1.35) | 1.20 (0.99-1.45) | 0.71 (0.40-1.24) |
| 50-64 | 1.27* (1.06-1.53) | 1.31** (1.09-1.57) | 1.00 (0.60-1.66) |
| ≥65 | 1.21* (1.00-1.46) | 1.21* (1.01-1.46) | 1.32 (0.76-2.29) |
| Race/ethnicity |  |  |  |
| White, non-Hispanic | Ref. | Ref. | Ref. |
| Black, non-Hispanic | 1.17** (1.04-1.31) | 1.14* (1.02-1.27) | 1.41 (0.94-2.11) |
| Hispanic/Latinx | 1.14* (1.03-1.27) | 1.12* (1.02-1.23) | 1.55* (1.04-2.33) |
| Asian, non-Hispanic | 0.84* (0.73-0.97) | 0.88 (0.74-1.04) | 0.34* (0.12-0.95) |
| American Indian/Alaskan Native, non-Hispanic | 0.89 (0.67-1.18) | 0.84 (0.64-1.11) | 1.31 (0.63-2.71) |
| Sex |  |  |  |
| Male | Ref. | Ref. | Ref. |
| Female | 1.03 (0.96-1.11) | 1.03 (0.95-1.11) | 1.00 (0.78-1.28) |
| Census region |  |  |  |
| Northeast | Ref. | Ref. | Ref. |
| Midwest | 0.91 (0.67-1.24) | 0.92 (0.67-1.25) | 0.79 (0.35-1.76) |
| South | 1.08 (0.80-1.46) | 1.07 (0.79-1.46) | 1.08 (0.53-2.21) |
| West | 1.06 (0.69-1.62) | 1.14 (0.74-1.74) | 0.43 (0.15-1.22) |
| **Health history** |  |  |  |
| Tobacco use |  |  |  |
| Current smoker | 1.01 (0.90-1.15) | 1.02 (0.90-1.16) | 1.07 (0.76-1.50) |
| Former smoker | 0.98 (0.89-1.09) | 0.98 (0.87-1.09) | 1.10 (0.86-1.42) |
| Never smoked | Ref. | Ref. | Ref. |
| Unknown | 1.21 (0.96-1.51) | 1.20 (0.97-1.48) | 1.49 (0.70-3.16) |
| Body mass index |  |  |  |
| Underweight | 0.99 (0.84-1.16) | 0.93 (0.77-1.14) | 1.16 (0.82-1.65) |
| Healthy weight | Ref. | Ref. | Ref. |
| Overweight | 0.98 (0.88-1.09) | 0.99 (0.87-1.13) | 0.85 (0.65-1.11) |
| Obesity | 0.97 (0.88-1.06) | 0.97 (0.88-1.08) | 0.94 (0.74-1.21) |
| Number of comorbidities |  |  |  |
| None | Ref. | Ref. | Ref. |
| 1-2 comorbidities | 1.07 (0.96-1.18) | 1.07 (0.96-1.19) | 1.01 (0.78-1.31) |
| 3-4 comorbidities | 1.10 (0.94-1.28) | 1.14 (1.00-1.31) | 0.70 (0.47-1.05) |
| 5+ comorbidities | 1.12 (0.82-1.52) | 1.06 (0.77-1.46) | 1.41 (0.85-2.33) |
| **Area-level characteristics (of patient’s residence zip code)** |  |  |  |
| Median household income |  |  |  |
| <$43,125 | 1.04 (0.83-1.29) | 1.06 (0.85-1.32) | 0.56 (0.27-1.14) |
| $43,125-$54,047 | 1.02 (0.87-1.20) | 1.04 (0.87-1.24) | 0.56 (0.26-1.17) |
| $54,048-$68,446 | 0.96 (0.82-1.14) | 0.99 (0.84-1.17) | 0.53 (0.27-1.04) |
| ≥$68,446.50 | Ref. | Ref. | Ref. |
| Percentage of population with only a high school degree |  |  |  |
| <25.5% | Ref. | Ref. | Ref. |
| 25.5%-33.7% | 0.93 (0.80-1.07) | 0.90 (0.77-1.05) | 1.70 (0.84-3.45) |
| 33.8%-41.2% | 0.95 (0.82-1.11) | 0.94 (0.80-1.10) | 1.46 (0.68-3.14) |
| ≥42.2% | 0.86 (0.69-1.07) | 0.87 (0.69-1.10) | 1.12 (0.55-2.30) |
| Percentage of population under age 65 without health insurance |  |  |  |
| <4.8% | Ref. | Ref. | Ref. |
| 4.8%-8.8% | 0.98 (0.82-1.16) | 0.98 (0.82-1.17) | 1.12 (0.66-1.90) |
| 8.9%-14.7% | 0.88 (0.69-1.12) | 0.89 (0.71-1.13) | 0.84 (0.50-1.42) |
| ≥14.8% | 0.87 (0.63-1.19) | 0.88 (0.63-1.22) | 0.86 (0.51-1.47) |
| **Cancer information** |  |  |  |
| Rural status of oncology practice |  |  |  |
| Urban | Ref. | Ref. | Ref. |
| Rural | 1.25* (1.01-1.55) | 1.41** (1.11-1.80) | 0.99 (0.71-1.38) |
| Cancer treatment type received or scheduled to receive (Ref.=Did not receive or was not scheduled to receive that treatment type) |  |  |  |
| Surgery | 1.77*** (1.49-2.09) | 1.71*** (1.41-2.07) | 3.03*** (1.60-5.71) |
| Radiation therapy | 1.13* (1.02-1.25) | 1.07 (0.97-1.19) | 2.02*** (1.39-2.93) |
| Drug-based therapy | 1.09 (0.92-1.30) | 1.12 (0.95-1.33) | 1.10 (0.70-1.71) |
| Transplant or cellular therapy | 1.72*** (1.37-2.16) | 1.50** (1.14-1.97) | 3.41*** (2.18-5.34) |
| Cancer type |  |  |  |
| Breast | Ref. | Ref. | Ref. |
| Hematologic/Blood | 1.34 (0.98-1.83) | 1.27 (0.92-1.76) | 2.35** (1.27-4.33) |
| Digestive/Gastrointestinal | 1.26*** (1.12-1.42) | 1.24*** (1.10-1.40) | 1.47* (1.08-2.01) |
| Lung | 1.07 (0.95-1.21) | 1.08 (0.93-1.25) | 0.94 (0.50-1.75) |
| Genitourinary | 0.93 (0.81-1.07) | 0.93 (0.80-1.09) | 0.91 (0.49-1.72) |
| Gynecological | 1.14 (0.95-1.36) | 1.14 (0.96-1.35) | 1.19 (0.65-2.17) |
| Other | 0.93 (0.78-1.10) | 0.97 (0.82-1.15) | 0.51* (0.30-0.88) |
| Extent of cancer |  |  |  |
| Local | Ref. | Ref. | Ref. |
| Regional | 1.25*** (1.12-1.39) | 1.20** (1.07-1.35) | 1.59* (1.07-2.35) |
| Metastatic | 1.30*** (1.13-1.49) | 1.27** (1.09-1.48) | 1.44* (1.01-2.04) |
| Cancer-free but receiving adjuvant therapy | 0.63** (0.46-0.87) | 0.63** (0.45-0.87) | 0.60 (0.23-1.56) |
| Unknown | 0.92 (0.66-1.28) | 0.93 (0.66-1.31) | 0.73 (0.41-1.28) |
| Cancer status |  |  |  |
| Progressing | Ref. | Ref. | Ref. |
| Stable | 0.98 (0.88-1.09) | 0.97 (0.86-1.10) | 0.88 (0.65-1.20) |
| Responding to treatment | 0.92 (0.77-1.11) | 0.93 (0.75-1.15) | 0.73 (0.42-1.26) |
| Unknown | 1.01 (0.82-1.25) | 1.05 (0.85-1.29) | 0.64 (0.40-1.04) |
| ECOG performance status |  |  |  |
| 0 | Ref. | Ref. | Ref. |
| 1 | 1.07 (0.97-1.19) | 1.06 (0.95-1.19) | 1.07 (0.80-1.42) |
| 2 | 1.02 (0.90-1.16) | 1.00 (0.87-1.14) | 1.00 (0.71-1.41) |
| 3+ | 1.19* (1.01-1.41) | 1.16 (0.99-1.37) | 1.86* (1.11-3.11) |
| Unknown | 1.05 (0.91-1.20) | 1.05 (0.90-1.23) | 0.83 (0.61-1.13) |
| Cancer diagnosis year |  |  |  |
| 2010 or earlier | Ref. | Ref. | Ref. |
| 2011-2019 | 1.07 (0.90-1.28) | 1.09 (0.91-1.31) | 0.80 (0.51-1.26) |
| 2020-2022 | 1.25** (1.07-1.46) | 1.29** (1.10-1.52) | 0.87 (0.55-1.37) |
| **COVID-19 information** |  |  |  |
| Patient developed any COVID-19 complications |  |  |  |
| No | Ref. | Ref. | Ref. |
| Yes | 1.16** (1.05-1.29) | 1.17** (1.06-1.29) | 1.12 (0.80-1.58) |
| COVID-19 severity |  |  |  |
| Uncomplicated | Ref. | Ref. | Ref. |
| Hospitalized, non-mechanically ventilated | 1.24** (1.09-1.42) | 1.23** (1.06-1.42) | 1.23 (0.95-1.58) |
| ICU admission, non-mechanically ventilated | 1.30* (1.06-1.59) | 1.31* (1.05-1.62) | 1.39 (0.65-2.95) |
| Mechanically ventilated | 1.59*** (1.30-1.95) | 1.54*** (1.25-1.90) | 2.51** (1.41-4.46) |
| COVID-19 case surge waves |  |  |  |
| First wave (March-June 2020) | 1.73** (1.20-2.50) | 1.78** (1.23-2.58) | 1.04 (0.45-2.38) |
| Second wave (July-November 2020) | 1.53** (1.17-2.02) | 1.62*** (1.23-2.13) | 1.10 (0.68-1.79) |
| Third wave (December 2020-March 2021) | 1.32* (1.06-1.64) | 1.35** (1.08-1.69) | 1.10 (0.79-1.54) |
| Fourth wave (April 2021-February 2022) | 1.25* (1.03-1.52) | 1.25* (1.02-1.52) | 1.09 (0.83-1.43) |
| Fifth wave (March-September 2022) | Ref. | Ref. | Ref. |
| N | 3428 | 2982 | 446 |
| **p* < .05, ***p* < .01, ****p* < .001. | | | |
| ^a^Adjusted prevalence ratios and 95% confidence intervals were computed using a multivariable Poisson regression with a log link function, adjusting for sociodemographic characteristics (rural status of patient residence, age group, race/ethnicity, sex, census region), health history (tobacco use, BMI, number of comorbidities), area-level characteristics (median household income, population with only a high school degree, population under age 65 without health insurance), cancer information (rural status of oncology practice, treatment type, cancer type, extent of cancer, cancer status, ECOG performance status, diagnosis year), and COVID-19 information (complications, severity, case surge wave time period). Standard errors were robust and clustered by the oncology practice. | | | |
